# Epidemiology of musculoskeletal injuries in Chinese collegiate dragon boat athletes: a cross-sectional survey

**DOI:** 10.3389/fpubh.2026.1830415

**Published:** 2026-06-08

**Authors:** Jingyi Wang, Huiru Ma, Ziwen Mu, Kazuhiro Imai, Hanyan Yan, Haoxiang Wang, Zhiqiang Han, Shaoshuai Shen, Xiao Zhou, Lin Yi

**Affiliations:** 1 School of Physical Education of Huazhong University of Science and Technology, Wuhan, China; 2 Department of Life Sciences, Graduate School of Arts and Sciences, The University of Tokyo, Tokyo, Japan; 3 School of Education and Welfare, Aichi Prefectural University, Nagakute, Aichi, Japan

**Keywords:** collegiate athletes, dragon boat, epidemiology, paddling phase, sports injury

## Abstract

This study aimed to investigate the epidemiological characteristics of injuries among Chinese collegiate dragon boat athletes. A cross-sectional retrospective survey was conducted in 2024 using electronic questionnaires distributed to collegiate dragon boat athletes nationwide. A total of 443 athletes (277 males and 166 females) who met the inclusion criteria were enrolled. Results showed that 115 athletes (26.0%) reported at least one injury in the previous year, with a total of 172 detailed injury events recorded. The overall injury rate was 1.57 per 1,000 athlete-exposures (AE) (95% CI: 1.34–1.81) and 0.43 per 1,000 training hours (95% CI, 0.36–0.49). Female athletes had significantly higher injury rates than males (2.05 vs. 1.29 per 1,000 AE, 0.56 vs. 0.35 per 1,000 training hours). Chronic injuries (57.6%) were more common than acute injuries (42.4%), with a chronic-to-acute ratio of 1.36:1. The shoulder (29.7%) and waist (23.8%) were the most common injury sites. Water training accounted for 64.5% of all injuries, with the pull phase (58.6%) and catch phase (36.0%) being the most frequently injury-associated technical phases. Most injuries (87.2%) were incidental or mild. These findings provide an epidemiological basis for developing targeted injury prevention strategies for collegiate dragon boat athletes, with emphasis on shoulder and waist protection during the pull and catch phases of paddling.

## 1 Introduction

Dragon boat racing originated in China, with a history spanning over 2000 years, evolving from ancient folk rituals and festival celebrations into a modern competitive sport (1). Athletes adopt a sitting position on the boat, driving the paddle with unilateral arm strokes. During the pull phase, the paddle blade pushes water backward; an equal and opposite reaction force is transmitted through the shaft and body to propel the boat forward (2). The paddling motion is characterized by continuous cyclicity which can be divided into four phases: catch, pull, exit, and recovery. The catch phase initiates the movement, where the blade enters the water at approximately 70°–80° relative to the water surface (nearly vertical), ensuring an efficient transfer of force from the paddler to the water, and the body flexes forward. The pull phase is the core of power output, generating the main propulsive force through lower limb pushing and trunk counter-rotation contralaterally, transmitting torque from the lower limbs through the core to the paddle shaft. During this phase, the top (outboard) shoulder undergoes extension and adduction, while the lower (inboard) arm produces elbow flexion; both wrists maintain a neutral position to transmit force efficiently through the paddle shaft. The exit phase serves as movement transition, with the blade leaving the water and the body posture recovering to the forward-lean, catch-ready posture, completing the stroke cycle. The recovery phase is for rest and reset, during which the blade swings back to the starting position in the air to prepare for the next cycle. The sequential connection of the four phases forms a power transmission process centered on the trunk musculature, including the erector spinae, multifidus, transversus abdominis, and internal and external obliques, which acts as a dynamic link between the lower limbs and the paddle, and also constitutes a potential site for injuries (3, 4). Accordingly, the lumbar spine (lower back), shoulder complex, knee, and wrist represent anatomical regions particularly vulnerable to injury during dragon boat paddling, consistent with upper-limb and trunk-dominant paddle sports.

One existing study reported an injury rate of 3.82 per 1,000 AE and 1.94 per 1,000 training hours among collegiate dragon boat athletes during training and competition; sex-specific rates were not separately reported (5). Another study on elite dragon boat athletes showed an injury rate of 10.19 per 1,000 AE (6). Regardless of competitive level, the injury rate was higher in females than in males. Among collegiate athletes, injuries were most common in the lower back (22.1%), shoulder (21.1%), and wrist (17.3%). Among elite athletes, the shoulder was the most common injury site (16.3%), followed by the hand (12.3%), lower back (11.2%), and pelvis (10.4%). Most dragon boat related injuries were chronic, with a chronic-to-acute count ratio ranging from 1.70:1 to 2.80:1 (5, 6); it should be noted that these values represent count ratios rather than formal incidence rate ratios. In a related paddling sport, Trevithick et al. reported that female rowers demonstrated significantly higher injury prevalence than males, at 35.0% versus 22.5%, highlighting that sex is a meaningful modifier of injury risk in paddling sports. The divergence in most-commonly injured regions between collegiate and elite cohorts likely reflects differences in cumulative training load, technique proficiency, and sport-specific conditioning; elite athletes typically sustain higher spinal loading volumes, predisposing the lumbar region to overuse injury.

To date, no studies have been conducted on the occurrence of injuries across different paddling phases in dragon boat racing. In other similar water sports, researchers have explored the relationship between paddling phases and injury mechanisms. Studies have shown that shoulder injuries in canoe athletes were closely related to high-load output during the pull phase, where the potential energy of each stroke was directly transmitted to the shoulder joint, and repeated cyclic loading was a major cause of high shoulder injury rates (7, 8). Biomechanical studies of rowing have found that the drive phase is when the lumbar spine bears the greatest compressive force, which is closely associated with a high incidence of lower back injuries. If athletes exhibit excessive trunk flexion with a “collapsed waist” at the catch position, the risk of lower back injury increases significantly (9, 10). Given the biomechanical similarities between rowing and dragon boat paddling, a comparable pattern of fatigue-induced lumbar flexion, analogous to the “collapsed waist” in rowers (9, 10), is highly suspected to occur in dragon boat athletes, particularly during the latter stages of prolonged training sessions when trunk stabilizer endurance is exceeded. Indeed, empirical evidence in dragon boat paddlers has highlighted a high prevalence of lumbar spine and shoulder injuries that significantly disrupt training capacity and sports participation (11).

Sports injuries not only directly affect training and competitive performance but may also cause persistent damage to athletes’ physical function. Therefore, clarifying the epidemiological characteristics of dragon boat related injuries is crucial for athletes, coaches, and relevant management authorities to develop scientific injury prevention strategies (7, 12). Especially for collegiate athletes in a critical period of academic development, injury prevention is a core prerequisite for ensuring training quality and prolonging sports participation. Existing dragon boat injury studies have mainly focused on athlete populations in countries such as Singapore and Iran (5, 6); however, no epidemiological investigation targeting Chinese collegiate dragon boat athletes has been found. This research gap limits the comprehensive understanding of injury characteristics and the development of targeted preventive measures for this population. Based on biomechanical evidence indicating peak joint loading during the catch (maximal reach) and pull (maximum propulsive force) phases, we hypothesized that these two phases would be associated with the highest injury frequency.

Accordingly, to improve injury prevention programs for collegiate dragon boat athletes, quantify injury differences among athletes with different training characteristics, and optimize rehabilitation pathways to improve performance and extend careers, this study aimed to: (1) investigate the injury incidence rate, injury prevalence, distribution characteristics, and most common anatomical sites of injury among collegiate dragon boat athletes; (2) identify high-risk injury sites, major injury types, and paddling phases associated with injury occurrence; and (3) compare injury incidence rates, injury prevalence, and anatomical distribution of injuries between male and female collegiate dragon boat athletes.

## 2 Methods

**Figure 1:**
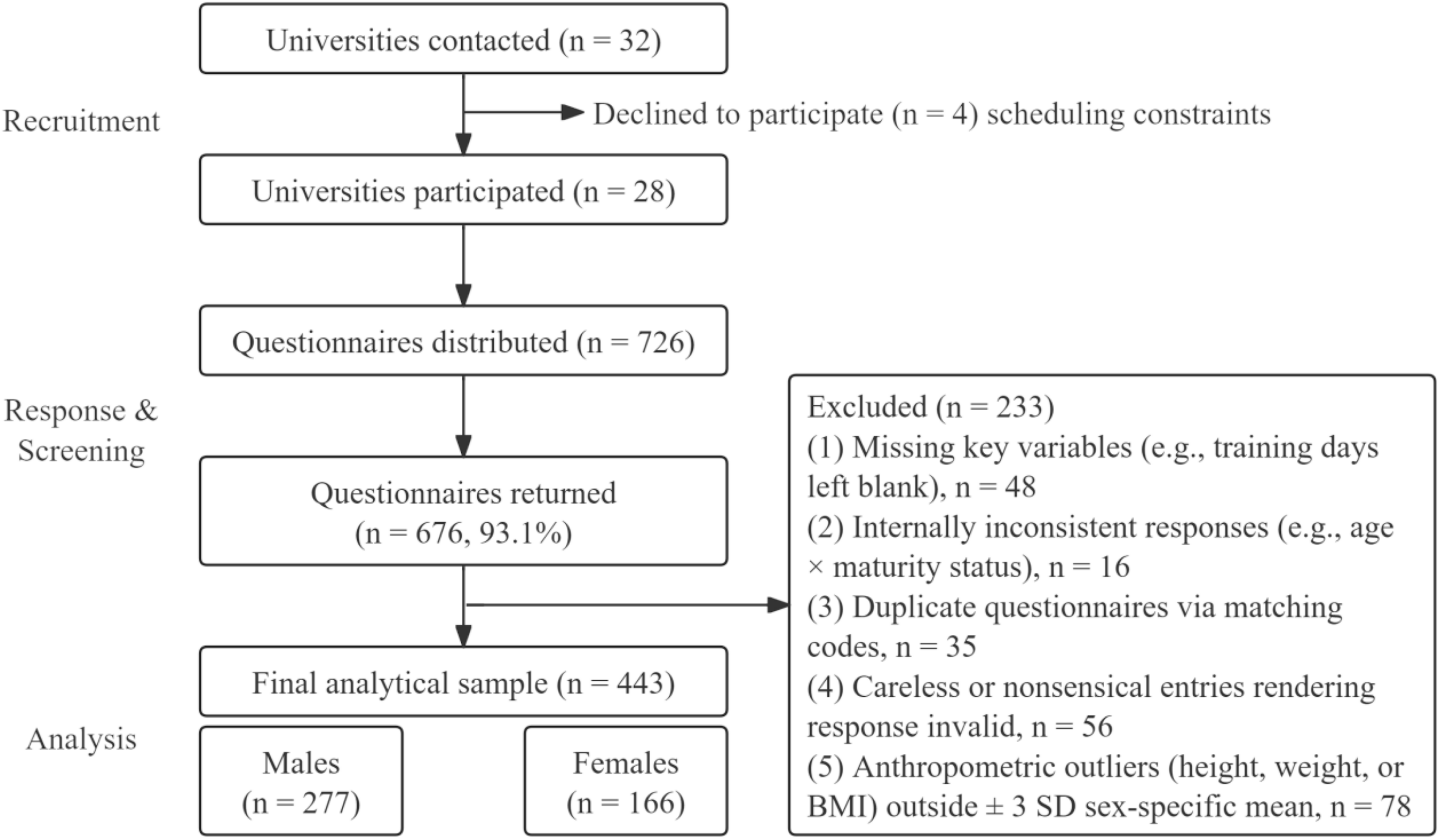
Participant flow diagram.

A cross-sectional retrospective survey was conducted in 2024. A total of 32 universities with registered collegiate dragon boat programmes were contacted; 28 agreed to participate (response rate 87.5%). The four non-participating institutions declined due to scheduling constraints. Questionnaires were distributed to all registered athletes at participating institutions (*n* = 726 in total) via online platforms and email. The survey collected data on sports injuries in the previous year. This study was approved by the ethics committee of the relevant institution, and all participants provided written informed consent. Of 726 questionnaires distributed, 676 were returned (response rate 93.1%). Following quality screening, 233 responses were excluded (see Figure 1), leaving 443 participants in the final analytical sample. The participant flow is illustrated in Figure 1.

The 28 participating universities were located across 12 provinces in China, spanning all major geographical regions (North, East, South, West, and Central China). To prevent duplicate responses, questionnaires were distributed by team coaches, one per registered athlete, using uniquely identifiable participant codes. No duplicate codes were detected during data cleaning.

Inclusion criteria were: (1) Current university student; (2) Regularly participated in dragon boat training during the survey period, with daily training ≥1 h and training frequency ≥3 days per week; (3) Voluntary participation with informed consent, completed questionnaire without outliers.

Participants were excluded for the following reasons: (1) missing key variables (e.g., daily training hours or weekly training days left blank), *n* = 48; (2) internally inconsistent responses (e.g., injury time-loss duration exceeding training history; severity coded as “time-loss” but zero days absence recorded), *n* = 16; (3) duplicate questionnaire submissions identified via matching participant tracking codes, *n* = 35; (4) careless or nonsensical entries rendering the response invalid (e.g., identical values entered for both height and weight, or random keyboard inputs), *n* = 56; and (5) anthropometric outliers (height, weight, or BMI falling outside ±3 SD of the sex-specific group mean), *n* = 78. In total, 233 responses were excluded, yielding a final analytical sample of 443 participants (277 males, 166 females).

The questionnaire was revised based on standardized definitions from the International Sports Injury Surveillance System and previous dragon boat injury research tools (5, 13), consisting of two parts. The first part collected basic information, including gender, age, height, weight, years of sports experience, daily training time, and weekly training days. The second part focused on recording dragon boat-related injuries in the previous year, including: (1) Injuries at 11 anatomical sites (shoulder, lumbar spine/waist, wrist, knee, chest, foot, thoracic spine/back, scapula, elbow, ankle, hip/buttock, and others); (2) Injury type (acute injury, chronic injury); (3) activity at the time of injury onset (water training, strength training, running training, competition, ergometer training, other); (4) Paddling phase during water training when injury occurred (catch, pull, exit, recovery); (5) Injury severity (classified by days absent from training or competition).

### 2.1 Definition of injury

A dragon boat injury was defined as any physical discomfort during training or competition that met any of the following criteria: any physical complaint in the musculoskeletal system resulting from dragon boat training or competition, regardless of whether it necessitated medical attention or caused absence from training (13, 14).

The questionnaire was designed to capture multiple injuries per athlete. Each injury event was treated as an independent observation for calculating injury counts and frequencies (such as injury sites, activity at the time of injury onset, paddling phases, and severity), consistent with standard injury surveillance methodology. For prevalence analyses (proportion of athletes injured), each athlete was counted only once regardless of the number of injuries sustained.

### 2.2 Injury severity

Injury severity was classified based on days absent from training or competition: (1) Incidental injury: no absence from training or competition (0 days); (2) Mild injury: absence for 1–7 days; (3) Moderate injury: absence for 8–28 days; (4) Severe injury: absence for more than 28 days (15).

### 2.3 Statistical analysis

The Shapiro–Wilk test was used to assess data normality. Normally distributed continuous variables were expressed as mean ± standard deviation (x̄ ± s), and categorical variables as frequency and percentage (%). Independent-samples t-tests were used to compare continuous demographic and training variables (e.g., age, height, weight, BMI, years of training, training hours) between males and females. The chi-square test or Fisher’s exact test was used to compare categorical variables (e.g., injury prevalence, acute vs. chronic injury distribution, injury sites, activity at the time of injury onset, paddling phases, and severity) between genders.

Athlete-exposure (AE) was defined as one athlete participating in one organized training session, consistent with the National Collegiate Athletic Association (NCAA) Injury Surveillance System definition. Annual AE was estimated as: number of athletes × weekly training sessions × 48 weeks, reflecting the full annual training cycle inclusive of pre-season, competitive season, and active recovery periods. The total calculated AE was 109,224 (males: 68,328; females: 40,896), and total training hours were 401,688 (males: 251,640; females: 150,048). Competition exposure was not included in the denominator owing to the inability to reliably quantify competition frequency via retrospective self-report; this constitutes a study limitation. Training time lost to injury was not deducted from the denominator, consistent with standard practice in retrospective surveillance studies.

Injury rates during dragon boat training were calculated as follows:

(1) Injury rate per 1,000 athlete-exposures (AE) = {*Σ* (total number of injuries)/Σ [(weekly training days × weeks)]} × 1,000; (2) Injury rate per 1,000 training hours = {Σ (total number of injuries)/Σ [(daily training duration × weekly training days × weeks)]} × 1,000.

Both metrics were reported because they serve complementary purposes. Athlete-exposures (per 1,000 AE) capture session-level risk and are useful for comparing studies with different session durations, whereas training hours (per 1,000 h) capture time-based risk and facilitate comparison with sports where session duration varies widely (e.g., rowing). Reporting both is consistent with recommendations in the International Olympic Committee consensus statement on sports injury surveillance [Bahr et al., (13)] and allows direct comparison with both AE-based and hours-based data in the existing dragon boat and paddling literature.

Injury rates were reported with 95% confidence intervals (CI) calculated using the Poisson distribution. Significant differences in injury rates per 1,000 AE and per 1,000 training hours between groups were assumed if 95% CIs did not overlap. A *p*-value <0.05 was considered statistically significant. All analyses were performed using SPSS 26.0 software.

## 3 Results

A total of 443 collegiate dragon boat athletes were enrolled, including 277 males (62.5%) and 166 females (37.5%). Table 1 shows the basic characteristics and gender differences of the participants. Male athletes were significantly taller and heavier than females (*p* ≤ 0.001), but no significant differences were observed in age, BMI, years of training, daily training time, weekly training days, or weekly training duration between groups (*p* > 0.05). Total training sessions were 68,328 for males and 40,896 for females; total training hours were 251,640 for males and 150,048 for females, with a total of 109,224 sessions and 401,688 h.

**Table 1:** Basic characteristics of collegiate dragon boat athletes.

Characteristic	Male (*n* = 277)	Female (*n* = 166)	*t*-value	*p*-value	Total (*n* = 443)
Age (years)	20.04 ± 1.70	19.96 ± 1.83	−0.421	0.674	20.01 ± 1.75
Height (cm)	177.76 ± 6.07	166.37 ± 5.96	−19.263	0.000**	173.49 ± 8.17
Weight (kg)	74.16 ± 8.52	64.51 ± 6.62	−3.355	0.001**	70.54 ± 8.34
BMI (kg/m^2^)	23.43 ± 2.13	23.31 ± 4.10	−0.090	0.929	23.39 ± 3.18
Years of training	2.00 ± 1.91	1.72 ± 1.35	−1.661	0.098	1.89 ± 1.72
Daily training time (hours)	3.26 ± 2.14	3.34 ± 1.76	0.420	0.675	3.29 ± 2.01
Weekly training days	5.14 ± 1.24	5.13 ± 1.51	−0.049	0.961	5.14 ± 1.35
Weekly training duration (hours)	18.93 ± 15.41	18.83 ± 12.47	−0.067	0.947	18.89 ± 14.36
Total training sessions	68,328	40,896	–	–	109,224
Total training hours	251,640	150,048	–	–	401,688

Measurement data are presented as mean ± standard deviation (x̄±s). Independent samples t-test was used for between-group comparisons. * *p* < 0.05 ** *p* < 0.01.

**Figure 2:**
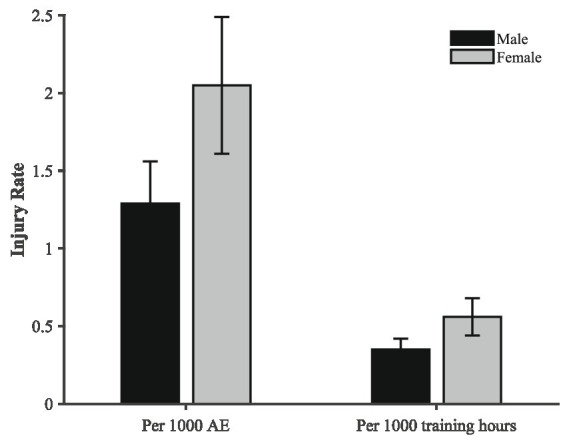
Comparison of injury rates.

Overall, 115 of the 443 athletes (26.0%) reported at least one sports injury in the previous year, with a total of 172 injuries recorded. The injury prevalence was significantly higher in females (62/166, 37.3%) than in males (53/277, 19.1%; χ^2^ = 6.37, *p* = 0.043). Of the 172 injuries, 73 (42.4%) were acute and 99 (57.6%) were chronic, yielding a chronic-to-acute count ratio of 1.36:1; chi-square analysis confirmed that chronic injuries were significantly more prevalent than acute injuries (χ^2^ = 4.21, *p* = 0.040). The proportion of chronic injuries was slightly higher in males (59.1%) than in females (55.9%), but this difference was not statistically significant (χ^2^ = 0.37, *p* = 0.425). The overall injury incidence rate was 1.57 per 1,000 AE (95% CI: 1.34–1.81). Female athletes had a significantly higher incidence rate (2.05 per 1,000 AE; 95% CI: 1.61–2.49) than male athletes (1.29 per 1,000 AE; 95% CI: 1.02–1.56). Similarly, per 1,000 training hours, the overall incidence rate was 0.43 (95% CI: 0.36–0.49), with females (0.56; 95% CI: 0.44–0.68) again showing significantly higher rates than males (0.35; 95% CI: 0.28–0.42) (Table 2; Figure 2).

**Table 2:** Injury prevalence, injury type, and incidence rates among collegiate dragon boat athletes.

Index	Male (*n* = 277)	Female (*n* = 166)	χ^2^	*p*-value	Total (*n* = 443)
Injured/total	53/277	62/166	6.37	0.043	115/443
Injury prevalence (%)	19.1	37.3	–	–	26.0
Total injuries	88	84	–	–	172
Acute injury *n* (%)	36 (40.9)	37 (44.1)	0.37	0.425	73 (42.4)
Chronic injury *n* (%)	52 (59.1)	47 (55.9)	–	–	99 (57.6)
Chronic-to-acute ratio	1.44:1	1.27:1	–	–	1.36:1
Per 1,000 AE (95% CI)	1.29 (1.02, 1.56)	2.05 (1.61, 2.49)	–	–	1.57 (1.34, 1.81)
Per 1,000 h (95% CI)	0.35 (0.28–0.42)	0.56 (0.44–0.68)	–	–	0.43 (0.36, 0.49)

Chi-square tests comparing the distribution of injuries by anatomical site between sexes showed no statistically significant difference overall (*p* > 0.05). However, site-specific analyses revealed that wrist injuries were significantly more common in males than in females (10.2% vs. 1.2%, *p* = 0.019), while knee injuries tended to be more common in females (9.5% vs. 2.3%, *p* = 0.052). No other significant sex differences were observed at individual anatomical sites (Table 3).

**Table 3:** Distribution of injury sites among collegiate dragon boat athletes by sex.

Site	Male (*n* = 88) *n* (%)	Female (*n* = 84) *n* (%)	Total (*n* = 172) *n* (%)	*p*-value
Shoulder	26 (29.5)	25 (29.8)	51 (29.7)	0.971
Lumbar spine/Waist	16 (18.2)	25 (29.8)	41 (23.8)	0.075
Wrist	9 (10.2)	1 (1.2)	10 (5.8)	0.019
Knee	2 (2.3)	8 (9.5)	10 (5.8)	0.052
Chest	5 (5.7)	3 (3.6)	8 (4.6)	0.722
Thoracic spine/Back	4 (4.5)	4 (4.8)	8 (4.6)	> 0.999
Scapula	5 (5.7)	2 (2.4)	7 (4.1)	0.444
Ankle	6 (6.8)	1 (1.2)	7 (4.1)	0.120
Elbow	2 (2.3)	4 (4.8)	6 (3.5)	0.432
Hip/buttock	3 (3.4)	3 (3.6)	6 (3.5)	> 0.999
Foot	1 (1.1)	0 (0.0)	1 (0.6)	> 0.999

Regarding activity at the time of injury onset, water training was the main setting, accounting for 64.5% (111 cases), followed by strength training (34 cases; 19.8%) and running training (14 cases; 8.1%). Injuries during competition accounted for only 1.7% (3 cases). Chi-square tests comparing the distribution of injuries by activity type between sexes showed no statistically significant difference overall (*p* > 0.05); however, the proportion of strength training injuries was higher in males (23.9%) than in females (15.5%) (Table 4).

**Table 4:** Distribution of injuries by activity at the time of onset among collegiate dragon boat athletes.

Scenario	Male (*n* = 88) *n* (%)	Female (*n* = 84) *n* (%)	Total (*n* = 172) *n* (%)
Water training	56 (63.6)	55 (65.5)	111 (64.5)
Strength training	21 (23.9)	13 (15.5)	34 (19.8)
Running training	7 (8.0)	7 (8.3)	14 (8.1)
Competition	2 (2.3)	1 (1.2)	3 (1.7)
Ergometer training	0 (0.0)	1 (1.2)	1 (0.6)
Other*	2 (2.3)	7 (8.3)	9 (5.2)
Total	88 (100.0)	84 (100.0)	172 (100.0)

**Figure 3:**
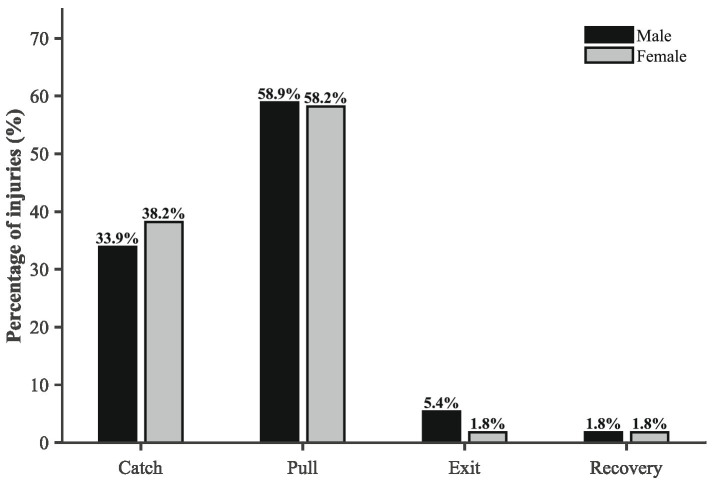
Distribution of injuries by paddling phase.

Of the 111 injuries during water training, 65 (58.6%) occurred in the pull phase, 40 (36.0%) in the catch phase, 4 (3.6%) in the exit phase, and 2 (1.8%) in the recovery phase. Chi-square test confirmed that injury frequency differed significantly across the four paddling phases (*p* < 0.001), with the pull and catch phases accounting for significantly more injuries than the exit and recovery phases (*p* < 0.05). No significant gender differences were observed in the distribution of injuries across paddling phases (χ^2^ = 1.106, *p* = 0.776) (Table 5; Figure 3).

**Table 5:** Distribution of injuries by paddling phase during water training.

Phase	Male (*n* = 56) *n* (%)	Female (*n* = 55) *n* (%)	Total (*n* = 111) *n* (%)
Catch	19 (33.9)	21 (38.2)	40 (36.0)
Pull	33 (58.9)	32 (58.2)	65 (58.6)
Exit	3 (5.4)	1 (1.8)	4 (3.6)
Recovery	1 (1.8)	1 (1.8)	2 (1.8)
Total	56 (100.0)	55 (100.0)	111 (100.0)

**Figure 4:**
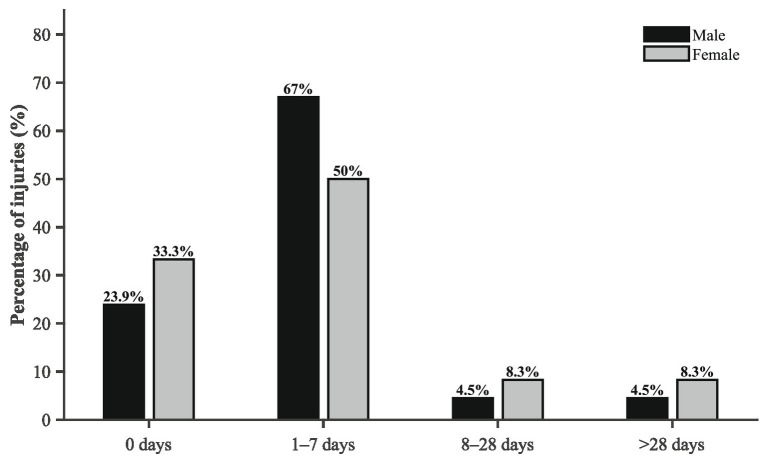
Distribution of injury severity.

Based on days of rest, 87.2% of all 172 injuries resulted in absence from dragon boat training for less than 1 week (incidental: 49 cases, 28.5%; mild: 101 cases, 58.7%). A total of 6.4% of injuries caused absence for 8–28 days, and 6.4% for more than 28 days. In both male and female athletes, the majority of injuries were incidental or mild. Although the proportion of severe injuries (more than 28 days of rest) appeared slightly higher in females (8.3%) than in males (4.5%), no significant gender differences were observed in overall injury severity (χ^2^ = 5.408, *p* = 0.144) (Table 6; Figure 4).

**Table 6:** Distribution of injury severity among collegiate dragon boat athletes.

Days of rest	Male (*n* = 88) *n* (%)	Female (*n* = 84) *n* (%)	Total (*n* = 172) *n* (%)
0 days (incidental)	21 (23.9)	28 (33.3)	49 (28.5)
1–7 days (mild)	59 (67.0)	42 (50.0)	101 (58.7)
8–28 days (moderate)	4 (4.5)	7 (8.3)	11 (6.4)
>28 days (severe)	4 (4.5)	7 (8.3)	11 (6.4)
Total	*88 (100.0)	*84 (100.0)	172 (100.0)

## 4 Discussion

This study provides the first comprehensive epidemiological profile of musculoskeletal injuries in Chinese collegiate dragon boat athletes, a population for whom no such data had previously been available. By establishing a robust baseline through a large-scale retrospective survey, this investigation fills a critical evidence limitation in the dragon boat injury literature, which has to date been limited to athlete cohorts in Singapore and Iran. The key findings: a substantial injury prevalence (41.1%), the predominance of chronic overuse injuries (57.6%), and the disproportionate frequency of injuries during the pull (58.6%) and catch (36.0%) phases of water training, collectively underscore the need for phase-specific, sex-tailored injury prevention strategies. The significantly higher injury incidence rates observed in female athletes (2.05 vs. 1.29 per 1,000 AE) further highlight the importance of sex-disaggregated analysis in informing targeted interventions.

Regarding injury rate, Mukherjee et al. (5) reported an injury rate of 3.82 per 1,000 AE and 1.94 per 1,000 training hours among collegiate dragon boat athletes in Singapore (*n* = 95), with higher rates in females (4.23 per 1,000 AE, 2.14 per 1,000 h) than in males (3.36 per 1,000 AE, 1.71 per 1,000 h). Zandi et al. (6) reported an injury rate of 10.19 per 1,000 AE among elite Iranian dragon boat athletes (*n* = 302), also with higher rates in females (10.73 per 1,000 AE) than in males (9.52 per 1,000 AE). Both studies reported higher injury rates in females, consistent with the present study: female injury rate (42.2%, 2.05 per 1,000 AE, 0.56 per 1,000 h) was significantly higher than male rate (40.4%, 1.29 per 1,000 AE, 0.35 per 1,000 h). However, the overall injury rate in this study (1.57 per 1,000 AE, 0.43 per 1,000 h) was markedly lower than those in Singapore and Iran. This discrepancy may result from several factors. First, different operational definitions of “injury” were used; for instance, this study employed a broad, self-reported definition encompassing any physical complaint regardless of medical attention or time loss, which may have captured more minor complaints and inflated the reported prevalence relative to studies using more restrictive, time-loss or medical-attention-based definitions. Second, the sample mainly comprised recreationally trained collegiate athletes with lower training intensity, volume, and professionalism; elite athletes in the Iranian study had significantly higher training loads and greater cumulative microdamage (6). Third, sample size differences may affect outcomes: the present study (*n* = 443) was much larger than the Singapore study (*n* = 95), improving stability and representativeness, whereas small samples may lead to greater variability in injury rates.

This study found that chronic injuries (57.6%) were the main type, with a chronic-to-acute ratio of approximately 1.36:1, consistent with Mukherjee et al. (5) (56.3% chronic). Dragon boat paddling is highly repetitive; athletes perform hundreds to thousands of cycles per training session, imposing cyclic loads on the shoulder girdle, spine, and upper limbs. Chronic injuries develop when tissue repair cannot match microdamage accumulation (14). Yang et al. (16) reported that chronic injuries predominate in low-contact collegiate sports. As a non-contact sport, dragon boat has few acute traumas, making chronic injuries the primary type.

Based on the high prevalence of chronic injuries observed in this study, several practical implications emerge for injury prevention and team management. First, coaches and sports medicine staff should systematically monitor athlete workloads, potentially utilizing the acute: chronic workload ratio, to identify periods of heightened risk for overuse injuries (17). Second, training programmes should incorporate periodized recovery weeks to facilitate adequate tissue adaptation and repair. Third, implementing regular musculoskeletal and movement screening is highly recommended to detect early signs of overuse pathology, particularly in high-risk anatomical sites such as the shoulder, lumbar spine, and knee (18). Finally, providing targeted education for coaches on progressive training load management and phase-specific biomechanical techniques is essential to mitigate injury risks and ensure the long-term health of collegiate dragon boat athletes.

The shoulder (29.7%) and waist (23.8%) were the most common injury sites, together accounting for 53.5% of all injuries, consistent with previous findings (5, 6). High shoulder injury rates are closely related to the biomechanics of dragon boat paddling. Ho et al. (15) performed a kinematic analysis showing that paddling involves repeated trunk flexion and rotation combined with multiplanar shoulder movement. Unilateral paddling creates asymmetric loading on the shoulder, leading to cumulative microdamage in rotator cuff muscles (14, 19). High waist injury rates relate to the seated paddling posture; with limited hip mobility in a seated position, the lumbar spine bears more rotational range of motion (20), contributing to high shoulder and waist injury rates in collegiate dragon boat athletes.

Sex differences were evident in the distribution of injuries across anatomical sites. Females demonstrated a higher proportion of knee injuries (9.5% vs. 2.3% in males) and waist injuries (29.8% vs. 18.2%), whereas males exhibited a higher proportion of wrist injuries (10.2% vs. 1.2% in females). The higher prevalence of knee injuries in females may be attributable to a larger Q-angle, which alters patellofemoral stress (21), and imbalanced quadriceps-hamstring strength, as reported in basketball and soccer (16, 22). The greater proportion of waist injuries in females may reflect relatively weaker upper limb and core strength, resulting in higher relative stress on rotator cuff and lumbar paraspinal muscles under asymmetric loading (11, 16). Conversely, the higher proportion of wrist injuries in males may stem from a higher proportion of strength training injuries (23.9% vs. 15.5%) and greater absolute grip strength (23), increasing cumulative wrist stress during paddling.

Regarding activity at the time of injury onset, water training accounted for 64.5% of injuries, followed by strength training (19.8%), with only 1.7% occurring during competition. It should be noted that these figures represent proportions of reported injuries by activity type rather than exposure-adjusted incidence rates, because activity-specific exposure time (e.g., total hours spent in water training vs. strength training) could not be reliably quantified from retrospective self-report. More injuries in training than competition were also observed by Mukherjee et al. (5), primarily due to much longer training exposure. Furthermore, athletes perform more thorough warm-ups and concentrate better during competition, while insufficient warm-up and fatigue accumulation are more common in daily training (12, 24).

The pull phase (58.6%) and catch phase (36.0%) accounted for 94.6% of water training injuries. It should be noted that these figures represent the relative frequencies of reported injuries by paddling phase rather than true exposure-adjusted risk estimates, because phase-specific exposure time (e.g., the exact time spent in each phase per stroke) could not be precisely quantified from the available data. The pull phase is the core power output stage, with peak shoulder and lumbar loads (15). Nonstandard techniques, such as over-reliance on upper limb strength instead of trunk rotation, cause abnormal local stress concentration. High injury rates in the catch phase may relate to impact loading when the blade enters the water, with the shoulder in a relatively unstable end-range position, increasing rotator cuff vulnerability (15). Based on these phase-specific injury characteristics, training should emphasize proper technique in these frequently injury-associated phases, promoting trunk rotation-driven upper limb movement. Specific strength training targeting these high-load phases, such as rotator cuff eccentric strength and core stability, should be integrated into daily conditioning (19, 20).

This study has several limitations. First, as a retrospective study, results depend on the accuracy of self-reported data, potentially introducing recall bias. Minor or short-term injuries may be underreported, underestimating injury rates. Second, the study may underestimate the prevalence of severe dragon boat injuries, as athletes with severe injuries may have been excluded due to inability to resume training. Third, injury data were based on self-reports rather than medical diagnoses, limiting verification of validity and detailed analysis of specific injuries (e.g., rotator cuff tear, disc herniation). Fourth, training load (e.g., intensity zones, power output) was not precisely quantified, restricting exploration of dose–response relationships between load and injury. Additionally, the relatively high exclusion rate (24.7%) due to strict screening and invalid entries is another limitation of this cross-sectional survey. Finally, because the study included only athletes currently training regularly, it may have excluded those who had discontinued training due to more severe injuries during the recall period. This potential “healthy worker effect” could bias injury estimates downwards, particularly underestimating the prevalence of severe injuries.

Future studies should prioritize prospective designs with objective workload monitoring (e.g., stroke-rate sensors) to establish dose–response relationships between training load and injury risk. Biomechanical studies linking phase-specific kinematics to injury aetiology are needed to confirm the mechanistic basis of the catch- and pull-phase injury predominance observed here. Randomized controlled trials evaluating phase-specific prevention programmes, such as shoulder pre-habilitation for females and lumbar stabilization training for males, would provide evidence for practice. Finally, similar surveillance efforts should be extended to elite and masters dragon boat athlete populations.

## 5 Conclusion

This investigation represents the most comprehensive injury surveillance study of Chinese collegiate dragon boat athletes to date. Female athletes sustained injuries at a rate approximately 70% higher than male athletes, underscoring the urgent need for sex-tailored prevention strategies, specifically upper-extremity pre-habilitation for female athletes. The identification of the pull and catch phases as the most frequently injury-associated paddling phases provides a mechanistic framework for biomechanics-informed coaching and technique modification. Chronic (overuse) injuries predominated, signalling the importance of workload management and progressive training periodization in injury prevention programmes. These findings equip coaches, sports medicine practitioners, and governing bodies with evidence to develop targeted injury surveillance and prevention infrastructure for this rapidly growing sport.

## Data Availability

The raw data supporting the conclusions of this article will be made available by the authors, without undue reservation.
